# Strong SIV gp120-specific IgG/IgA responses in milk of African green monkeys may contribute to the rarity of postnatal transmission in this species

**DOI:** 10.1186/1742-4690-9-S2-P198

**Published:** 2012-09-13

**Authors:** JD Amos, AB Wilks, GG Fouda, SD Smith, GR Overman, K Beck, MA Moody, GD Tomaras, SR Permar

**Affiliations:** 1 Duke Human Vaccine Institute, Durham, NC, USA; 2 Division of Viral Pathogenesis, Beth Israel Deaconess Medical Center, Boston, MA, USA; 3 Division of Laboratory Animal Resources, Duke University, Durham, NC, USA

## Background

African green monkeys (AGMs), natural SIV hosts, sustain nonpathogenic infections and rarely transmit the virus to their suckling infants, despite exposure to high milk virus RNA loads. Furthermore, we previously reported strong autologous neutralization responses in milk of SIV-infected AGMs which could contribute to impediment of infant virus acquisition. Comparing mucosal B cell populations and responses in milk of AGMs to that of rhesus monkeys (RMs), symptomatic SIV hosts with high rates of postnatal transmission, could elucidate the protection against postnatal virus transmission in natural SIV hosts.

## Methods

Six female AGMs and four female RMs were hormonally-induced into lactation prior to intravenous inoculation with SIVsab92018 and SIVmac251, respectively. B cells in milk and blood were phenotypically analyzed by flow cytometry. Total and SIV gp120-specific IgG/IgA responses in milk and plasma were measured using autologous virus-specific ELISA.

## Results

SIV gp120-specific IgG responses were approximately one log higher in milk (p=0.02) and plasma (p=0.009) of AGMs compared to that of RMs. Remarkably, the milk SIV gp120-specific IgA response of AGMs was two logs higher than that of RMs (p=0.009). Comparing the milk SIV gp120-specific IgA responses to other mucosal compartments of AGMs, milk responses were higher than rectal (p = 0.03), but similar to vaginal responses. Although there were no significant differences in the milk memory B cells populations of AGMs and RMs, we observed a reduced proportion and absolute number of naive B cells (CD20+, IgD+, CD27-) in milk of RMs (median = 7.9%, 0.16 cells/µl) compared to AGMs (median = 26.5%, 4.2 cells/µl) (p = 0.009 and 0.06, respectively) during chronic infection.

## Conclusion

AGMs appear to preserve SIV-specific IgG/IgA responses in milk during chronic infection, potentially due to a lack of immune activation and B cell dysfunction, which may contribute to the rarity of postnatal SIV transmission in this natural host species.

